# Single Echo MRI

**DOI:** 10.1371/journal.pone.0086008

**Published:** 2014-01-21

**Authors:** Gigi Galiana, R. Todd Constable

**Affiliations:** 1 Department of Diagnostic Radiology, Yale University, New Haven, Connecticut, United States of America; 2 Department of Biomedical Engineering, Yale University, New Haven, Connecticut, United States of America; University of Minnesota, United States of America

## Abstract

**Purpose:**

Previous nonlinear gradient research has focused on trajectories that reconstruct images with a minimum number of echoes. Here we describe sequences where the nonlinear gradients vary in time to acquire the image in a single readout. The readout is designed to be very smooth so that it can be compressed to minimal time without violating peripheral nerve stimulation limits, yielding an image from a single 4 ms echo.

**Theory and Methods:**

This sequence was inspired by considering the code of each voxel, i.e. the phase accumulation that a voxel follows through the readout, an approach connected to traditional encoding theory. We present simulations for the initial sequence, a low slew rate analog, and higher resolution reconstructions.

**Results:**

Extremely fast acquisitions are achievable, though as one would expect, SNR is reduced relative to the slower Cartesian sampling schemes because of the high gradient strengths.

**Conclusions:**

The prospect that nonlinear gradients can acquire images in a single <10 ms echo makes this a novel and interesting approach to image encoding.

## Introduction

Parallel imaging, the use of locally sensitive receivers to provide encoding, typically aims to reduce scan time by reconstructing images from a reduced number of timepoints.[1]–[11] To facilitate this, several groups, including our own, have studied whether the geometry of gradient encoding can better complement the information from locally sensitive receivers. [12]–[17] Previous work has looked at whether this allows a further reduction in the minimum number of echoes due to increased encoding efficiency when the encoding is shared between the magnetic field gradients and the receiver coil sensitivity profiles. This has led to many studies of image encoding with nonlinear gradient shapes, producing good image reconstructions from highly undersampled datasets. [12]–[14] Previous nonlinear gradient encoding work has also explored single shot trajectories employing EPI-like readouts. One example is 4D-RIO (4-Dimensional Radial In/Out) which uses an offset radial acquisitions on both linear and nonlinear gradient channels. [18] EPI-like readouts have also been combined with trajectories designed to enhance or sacrifice resolution in different parts of the field of view. [19]

To date, these nonlinear fields have primarily had constant amplitude over the readout. Here we present an image encoding strategy that can produce extremely short image acquisition times by using time varied waveforms that collect a single echo, making a 64^2^ image feasible in 4 ms. The proposed trajectories impose a unique timecourse on each pixel in the image by applying rotating nonlinear fields. Pixels with redundant or similar timecourses have an azimuthal symmetry that can be distinguished via receiver coil encoding.

Rather than producing images from a reduced number of timepoints, the trajectory is designed to minimize the total slew of the acquisition, allowing for high bandwidths that acquire the N^2^ datapoints in a minimal amount of time. [20]–[22] These images produce a full 2D image after playing out a single sinusoidal waveform on two nonlinear gradient channels. Thus, the entire trajectory can theoretically be compressed to a 4 ms acquisition time without violating peripheral nerve stimulation (PNS) thresholds.

### Theory

The shift in point of view that motivated this family of trajectories was to consider gradient encoding as a means of applying a unique phase/magnitude code or signature to each pixel in the image. We refer to this timecourse as the code of that pixel. The code of a pixel at (x,y) can be written as: 
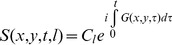
(1)where *l* is the index for coil sensitivity *C_l_*. So long as the code of a pixel is unique and the noise is sufficiently low, the magnitude of signal coming from that pixel can be deduced.

We can further justify this assertion by connecting it to the matrix methods that are increasingly used to reconstruct MR images. Typically, the reconstruction problem is defined as the solution, *x*, that satisfies: ***Ax*** = ***b***, where *b* is the measurement data, *A* is the encoding matrix, and *x* is the object or image. [23]–[30] One typically describes *A* as being assembled row by row. Each row describes the encoding *A_i_* being applied when we measured datapoint *b_i_*. Thus, each row captures the equation: ***A_i_x*** = ***b_i_***

However, we can just as easily describe this same encoding matrix as being assembled column by column. Each column describes the code followed by a given pixel over the full course of the acquisition. From this perspective, the matrix equation can be understood as a decomposition of different timecourses, like those commonly used in fMRI. [31], [32] The object vector is equivalent to the weight vector solved for in fMRI studies (the task for example in such studies), and the weight of signal that follows a given code tells us the density of spins in a given pixel.

Requiring a unique code for each pixel is equivalent to requiring that the columns of the encoding matrix be linearly independent to resolve the image. If two pixels have identical codes due to gradient evolution (i.e. if two columns are identical), those pixels “fold” on top of each other. However, just as in SENSE, these pixels can be unfolded so long as the coil weightings on the folded pixels are not identical. In terms of the encoding matrix, the coil weightings create differently weighted versions of the timecourse code, so that previously identical columns become linearly independent.

The purpose of such sequences is to impose a unique code on each voxel. Such a scheme should therefore be designed such that voxels with the same gradient code should be distant in space so that the receiver coil weightings may distinguish them. Furthermore, to achieve very fast acquisitions, we have focused on very smooth gradient waveforms that could be played out in a single acquisition window.

**Figure 1:**
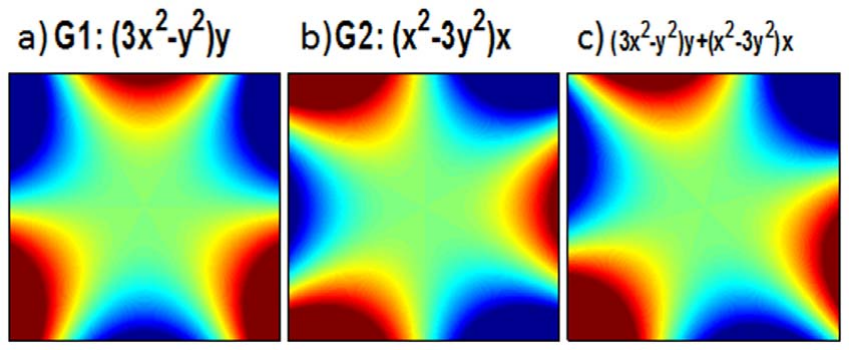
Third Order Spherical Harmonics. Any orientation of this field can be generated by the appropriate linear combinations of G1 and G2. Playing G1 and G2 with cosine and sine modulation respectively generates a field rotating with constant angular velocity.

One field that produces an interesting code is that of a rotating *l* = 3, |*m*| = 3 spherical harmonic, as shown in Figure 1. In practice, any rotated version of that field can be achieved by playing different proportions of the following third order fields: 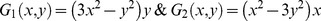
(2)as shown in panels 1(a)–(c). If we play *G*_1_ with a cosine amplitude modulation and *G*_2_ with a sine amplitude modulation, we rotate the field through π/3, which returns us to the initial orientation and spins anywhere in the FOV are dephased and rephased in this cycle.

**Figure 2:**
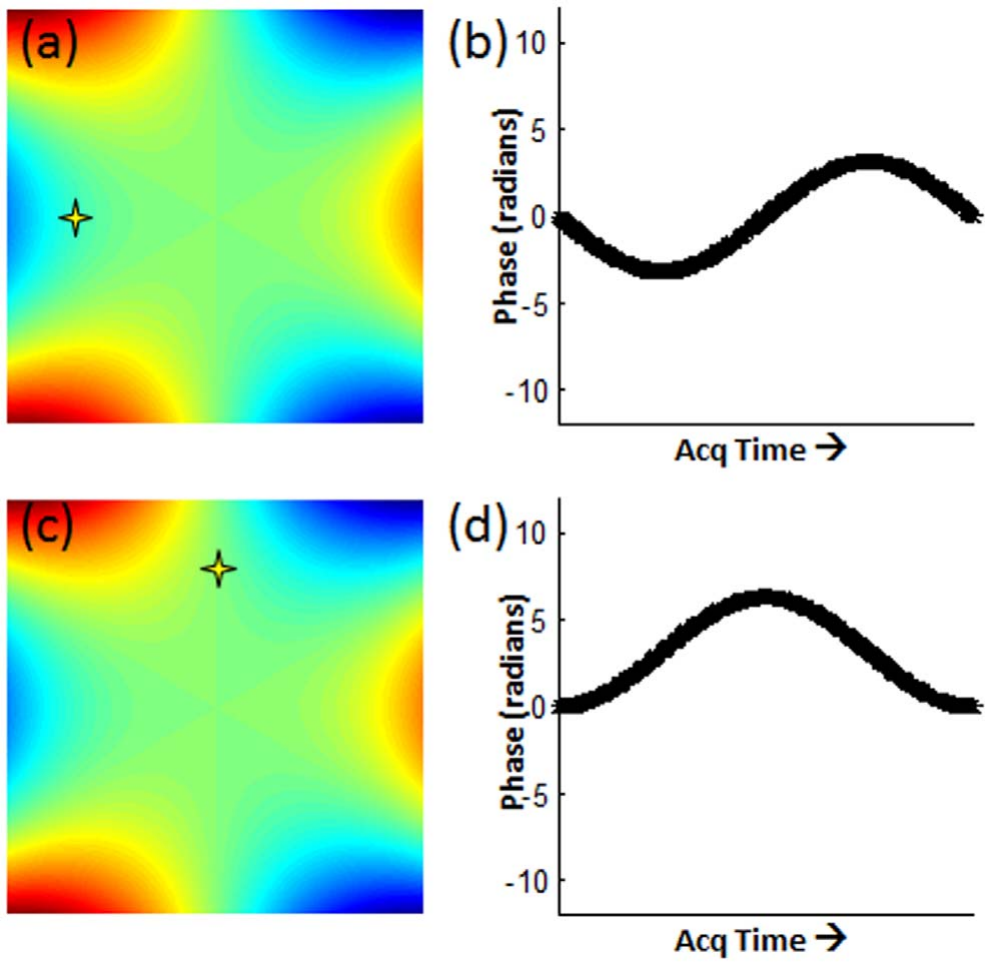
Code for Peripheral Voxels under a Rotating Third Order Field. Evolving under a rotating version of the field shown in (a), the highlighted voxel accumulates a sinusoidal phase that returns to zero over a full π/3 rotation of the field. A voxel at the same radius but a different angular position has the same amplitude of variation, but follows a sinusoidal phase code with different phase.

Figure 2b shows the code of the marked pixel (Figure 2a) evolving under this field as it rotates clockwise. The pixel begins accumulating negative phase at a rapid rate, but as rotation continues, this rate decreases to zero as the null region of the field approaches the pixel. Next the pixel begins to accumulate positive phase, which it continues to do as the positive lobe of the field passes over it. The pixel reaches its maximum positive phase when the next null reaches it, and it then rephases to its initial phase as it is traversed by the next half of the blue lobe.

We can also solve for this phase code analytically. The gradient as a function of time is: 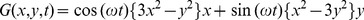
(3)where *t* samples each dwell time of the acquisition and *ω* is 2π divided by the total acquisition time. Then the argument of the exponential in Equation 1, the phase code of a voxel at (*x,y*), is: 
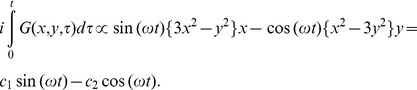
(3)


Thus, over the entire rotation, the phase code of any pixel will look like a sine wave. The amplitude of the sine wave will be proportional to its distance from the center, and the starting phase is related to its azimuthal position. (See Figures 2c and 2d for the location and code of a voxel at the same radius but different azimuthal position.) Furthermore, every voxel returns to its initial phase over the entire trajectory, so this is a self-refocused code. Voxels at the same radius and π/3 radians apart will receive the same phase code, but these are easily distinguished by the receiver coil sensitivity encoding, especially at the periphery of the FOV.

**Figure 3:**
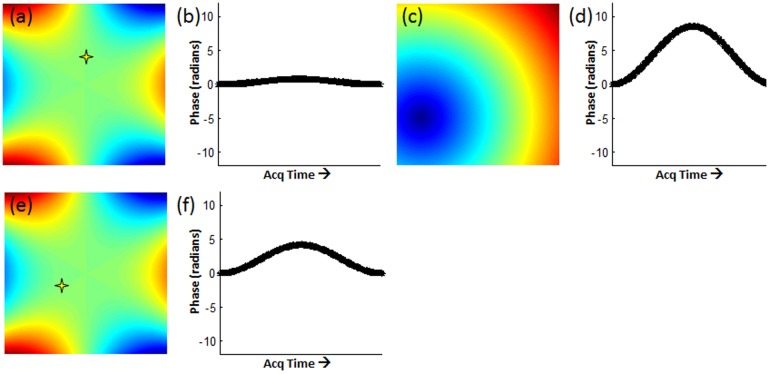
Code for Central Voxels. Evolving under just the rotating third order field, a voxel such as that marked in (a) has little phase variation over the acquisition. This is remedied by adding an offset 2nd order field like that shown in (b), using a combination of x, y, and z2 gradient shapes. Giving this field a cosine amplitude modulation, the relatively flat part of the gradient changes both position and shape during the acquisition. This creates the phase code seen in (d) for the same voxel. Voxels close to the nominal center placement, like the voxel highlighted in (e) also shows a significant phase code (f) because the flat part of the field moves during the acquisition.

Of course the weakness of this simple code is that the amplitude of the sine wave diminishes near the center of the field of view, which is also where coil encodings become least distinct. This can make voxels difficult to distinguish, as can be seen in Figure 3. Here we show a voxel closer to the center of the FOV (3a) and its timecourse (3b).

To address this limitation, we added a field with the shape of (x−x_0_)^2^ + (y−y_0_)^2^, with x_0_ = −.45FOV and y_0_ = −.19FOV. This field was then played out with an amplitude modulation that was out of phase with field *G*_1_ and in phase with field *G*_2_. The spatial shape of this additional field, which in practice is created by a linear combination of the x, y, and z^2^- ½(x^2^+y^2^) gradients, is shown in Figure 3c, and is very similar to the fields used in O-space imaging [12], [13]. In general, adding linear components to the z2-gradient only shifts the location of the field's flat region or center. However, because the strength of the linear magnetic gradient fields changes during acquisition, the flat region of the composite field across the field of view is translated across the field of view during the readout. Though the relative proportions of G_z2_, G_x_, and G_y_ remain constant, which corresponds to a constant center placement of the G_z2_ field, the changing proportion of G1 causes the saddle of the composite field to shift during the readout. Simultaneously, the changing proportions of nonlinear gradients also cause variations in the topology of the field, from a pure G1/G2 combination, like those shown in Figure 1, to a stretched out version that results from adding z^2^- ½(x^2^+y^2^) gradient. The z^2^- ½(x^2^+y^2^) term is offcenter and thus introduces a steep gradient across the center of the FOV where the G1/G2 gradients are flattest thus allowing us to resolve spins in the center of the FOV.

Figure 3d shows the phase accumulation of this same voxel (3a) using the modified trajectory. Since this additional field could increase the overall bandwidth of the acquisition, the third order fields are scaled accordingly to maintain the same bandwidth. In addition, we show a voxel closer to the valley of the additional field (3e) and its code (3f). These voxels now both have significant and distinct phase excursions that can be used to distinguish their codes.

## Methods

**Figure 4:**
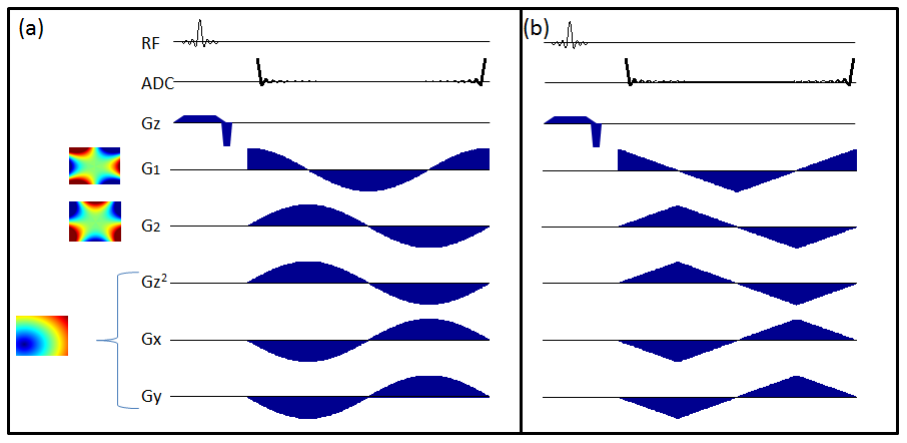
Pulse Sequence for Single Echo Imaging. The sinusoidal variations on the third order channel create a rotating third order field. Meanwhile, since the amplitude modulation on the 1st and 2nd order fields is different, the flat part of the gradient shifts in space, and the center of the FOV is fully encoded. These trajectories encode the entire image in a single shot, and the minimum slew analog on the right could yield a complete image from a single 4

Images were encoded using rotated versions of the fields shown in Figure 1. This was simulated by applying linear combinations of fields like those shown in Figure 4. Readout sampled 4096 points, and encoding was simulated in Matlab at a 64^2^ matrix size. Noise was added to each readout from a white Gaussian distribution, which was adjusted to provide an SNR of approximately 100 for Cartesian images. The simulations used experimental receiver coil profiles from an 8 channel coil. To capture intravoxel dephasing at the edge of the field of view, we calculated the encoding matrix at a resolution of 256^2^ and then summed over the 4×4 square corresponding to our coarser voxels. This downsampled encoding matrix was used for both data generation and image reconstruction. Reconstruction was performed with a Kaczmarz algorithm with 5 iterations. [12], [23] The reconstruction included both gradient and coil encoding in a single matrix, making this a true parallel imaging reconstruction.

To study higher resolution reconstructions, we also simulated reconstructions with longer acquisition times and higher reconstruction matrix sizes. For 128^2^ images, the encoding matrix was again 4-fold oversampled in space and binned down to capture intravoxel dephasing, and noise was added by the same procedure. Longer acquisitions were explored several ways: 1) by considering the same shapes and number of points but with twice the dwell time, leading to a longer acquisition window 2) by considering the longer acquisition window, but maintaining the dwell time, leading to twice the number of points, and 3) by acquiring more than one echo. In the two echo simulations, the first echo uses linear and second order fields that create G(x,y) =  (x−x_0_)^2^ + (y−y_0_)^2^, while the second echo uses the field G(x,y) =  (x+x_0_)^2^ + (y+y_0_)^2^. The amplitude modulation and the higher order gradient shapes are otherwise the same.

To study the point spread function (PSF) properties of the reconstruction, we also performed reconstructions of single point images for each position in the FOV using our simulation for the sinusoidal trajectory. These were also performed at 64^2^ resolution, with noise adjusted to produce an SNR of 150, and using experimental coil receiver sensitivity profiles which were windowed to the diameters of the bore of the coil before being included in the reconstruction matrix. For each single point image, we calculated a metric reflecting the localization of the PSF by calculating the signal intensity at the occupied voxel divided by the mean of signal intensity at all other voxels in the image.

## Results

Figure 4a shows the basic pulse sequence for the rotating field trajectory, with cosine and sine amplitude modulations on the gradients. Limiting the gradient slew rate to 45T/s over the 20 cm field of view (a limit commonly imposed on clinical scanners), we find that this readout, which acquires the entire image, can be played out in 8.2 ms. An acquisition time this short could be achieved with the following maximum amplitudes for each field: 1800 Hz/cm, 180 Hz/cm^2^, and 1400 Hz/cm^3^ for the 1^st^, 2^nd^, and 3^rd^ order fields, respectively. Because fields are steepest at the edge of the field of view, imaging over larger areas would require lower bandwidth acquisitions and longer readouts, but our projections would apply to normal brain imaging using a head insert to generate the nonlinear fields.

To test the limits of fast imaging with this sequence, we also studied a trajectory like that shown in Figure 4b. This linearized trajectory reduces the maximum slew rate on each channel and would theoretically allow us to acquire the data in 4.3 ms without exceeding the mean threshold for peripheral nerve stimulation discomfort (55T/s) over a 20 cm field of view. [33] This acquisition scheme would imply a dwell time of approximately 1 µs. Maximum amplitudes along each channel would need to be approximately doubled to achieve the same encodings over these shorter dwell times.

Note that in both these pulse sequence diagrams, the sequences and signals are shown in an arrangement that produces codes like those shown in Figures 2 and 3. Thus the signal begins and ends at its fully rephased state, reflecting the analysis described in the Theory section. However, a symmetric echo can easily be achieved with little additional time penalty. In practice, this could be accomplished by simply running the sinusoidal trajectories for an extra half-cycle and reading out the data in the last two thirds of the trajectory. This would also place the echo at the center of the readout, which is typically the best way to collect an echo.

**Figure 5:**
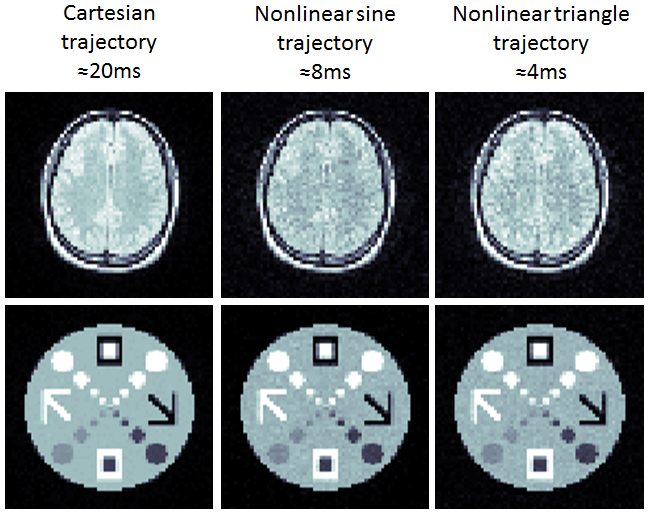
Simulation Results. Using two phantoms, we compare 64^2^ reconstructions acquired with a standard Cartesian trajectory, and compared these images with those obtained using the two trajectories diagrammed in Figure? 4. Both the sinusoidal trajectory and lower slew rate analog give excellent reconstructions, though dephasing notably degrades SNR. However, these are promising reconstructions for single echo data that could theoretically be acquired in as little as 4 ms.

Figure 5 shows reconstructions of images simulated from acquisitions with both the sinusoidal and triangular trajectories described above. The first column shows two phantoms reconstructed using a standard Cartesian sampling scheme. If acquired in an echo planar acquisition, this would correspond to a minimum acquisition time of approximately 20 ms. The next two columns show these phantoms as reconstructed from the trajectories shown in Figure 4a and 4b, respectively. These images show good image reconstruction, though noise levels are higher than in the corresponding Cartesian trajectory. However, these images could be generated by a single echo that is extremely short and potentially allowing unprecedented temporal resolution.

**Figure 6:**
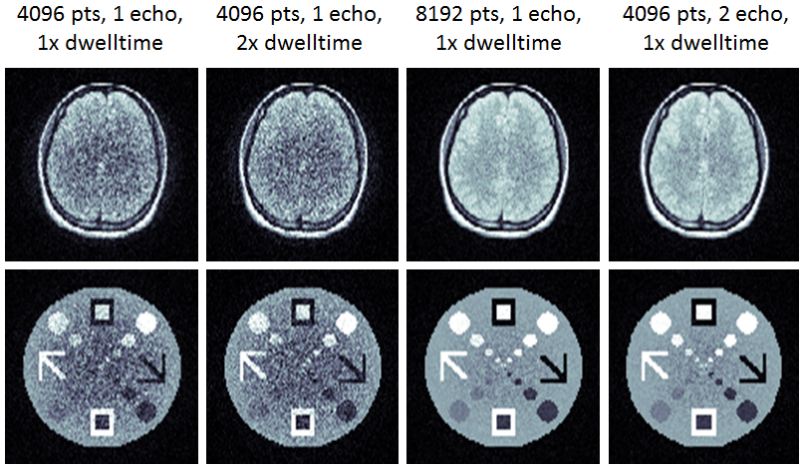
Higher Resolution Simulations. With the same trajectory and acquisition used in Figure? 5, but reconstructing to a 128^2^ matrix size, (first column) we find a notable lack of resolution, particularly at the center. Doubling the length of the acquisition window, but acquiring the same number of points (2^nd^ column) shows little improvement, presumably due to incoherent artifacts related to inadequate sampling bandwidth. However, by doubling the length of the acquisition window and collecting twice as many points (third column), the images are greatly improved. We also simulated the effect of using a two-echo acquisition (fourth column), but little image quality is gained with the additional repetition time.

To achieve higher resolution reconstructions, we studied various trajectory modifications, with those results shown in Figure 6. The first column shows reconstructions of the same data used to reconstruct the 64^2^ images of Figure 5 but here used to reconstruct a 128^2^ image. The next two columns show reconstructions of acquisition windows that would be twice as long. The longer acquisition window would either result from acquiring the same number of points, but doubling the dwell time, as we show in the second column. This is equivalent to doubling the gradient strength but, relative to the aforementioned acquisitions run at maximum slew, this could only be achieved by increasing dwell time. In the third column, the longer acquisition uses the original gradient strengths and dwell times but acquires twice the number of points. This shows a significant improvement in the reconstruction. Finally we consider acquiring twice the original data over two repetitions of the sequence. In the second repetition, we use a different (x_0_,y_0_) for the circularly symmetric field, but this shows little improvement over the longer readout and loses the advantages of acquiring the entire image in a single echo.

**Figure 7:**
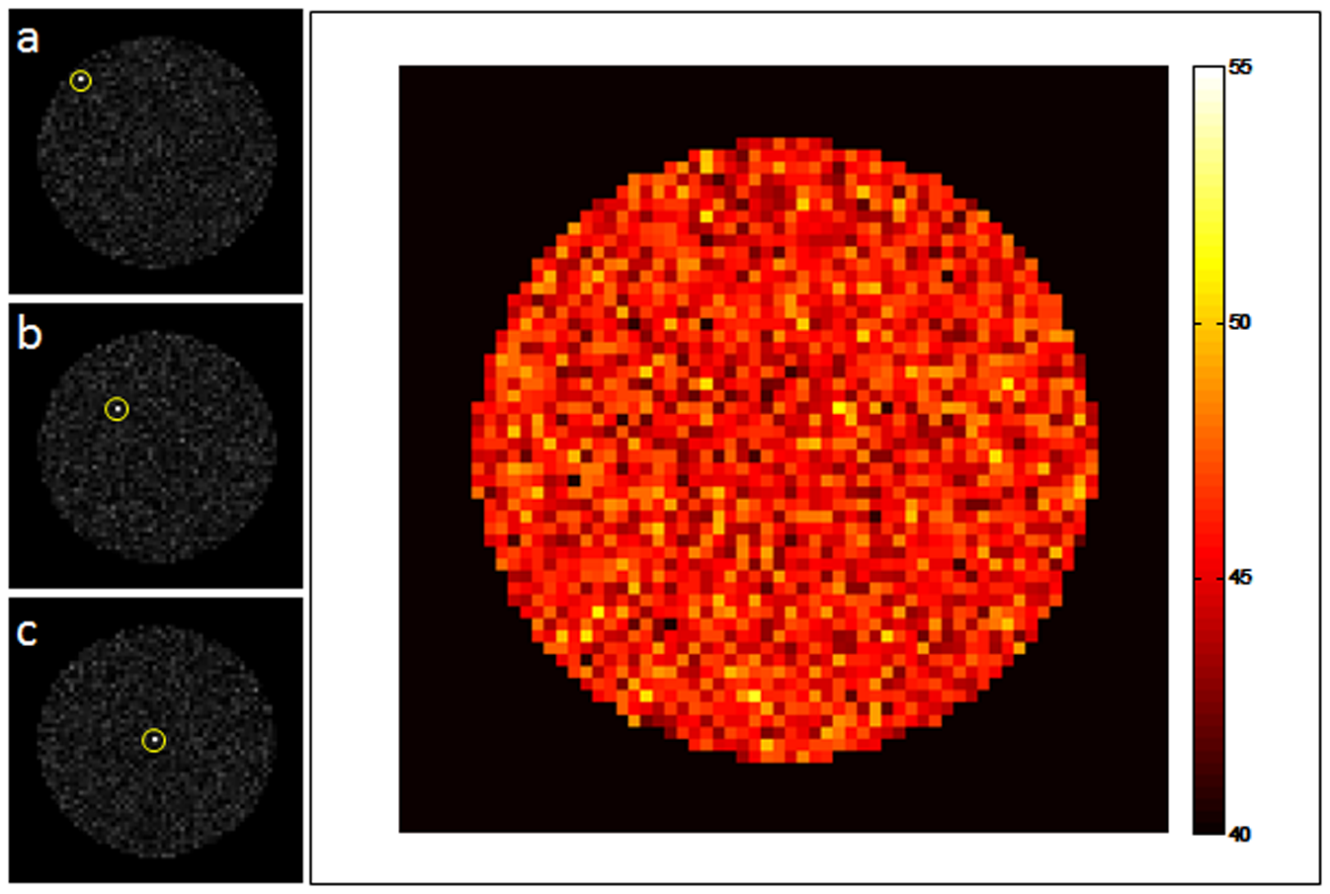
Point Spread Function Map. Using the proposed 8(a–c) as well as a map of (signal at target voxel)/(mean signal in all other voxels), shown in (d). As can be seen from the individual single point images, the proposed encoding results in noise-like artifacts distributed throughout the image, leading to a relatively uniform ability to localize signal across the field of view.

In addition, we have also calculated and mapped a metric reflecting our ability to localize signal in different parts of the field of view. Figure 7 shows three individual single point image reconstructions along with a map reporting the intensity observed at the target voxel divided by the average intensity in all other voxels. As can be seen in panels a–c, the proposed trajectory results in highly incoherent noise-like artifacts but no blurring. These trajectories result in a relatively uniform sensitivity across the field of view.

## Discussion

Dynamically changing the gradient amplitude in sequences that use nonlinear gradients can open a complex parameter space that makes sequence design challenging. Due to memory constraints and the structure of our simulation program, our simulations were only feasible to a 128^2^ resolution, but the finer images could be reconstructed by doubling the number of sample points in a single echo. Still higher resolution reconstructions may also require only an extended readout, still acquiring data for the entire image in a single short echo.

By focusing on the code of each voxel, we can design sequences that encode the entire image in a single readout. Redundancies in the code are kept spatially separated such that the coil sensitivity profiles from parallel receiver arrays can resolve the resulting ambiguities. While the examples shown here used third order gradients combined with an off-center z2 gradient a wide-range of gradient shapes could potentially be explored to yield single echo images. The important feature is to design dynamic gradient trajectories that impose a unique phase and frequency history on each voxel in the FOV in order to spatially localize each voxel. This approach abandons the constraints that Fourier transform theory conventionally imposes in terms of the data required to reconstruct an image and yields a highly efficient encoding strategy.

The use of extremely smooth sinusoidally varying gradient amplitudes may make it possible to play these out on very short time scales without violating physiological peripheral nerve stimulation limits. Using these techniques, our simulations suggest that it may be feasible to acquire a 64^2^ image in as little as 4 ms using only a single echo acquisition.
